# Ovarian hyperstimulation in premenopausal women during adjuvant tamoxifen treatment for endocrine-dependent breast cancer: A report of two cases

**DOI:** 10.3892/ol.2014.2319

**Published:** 2014-07-04

**Authors:** CLELIA MADEDDU, GIULIA GRAMIGNANO, PARASKEVAS KOTSONIS, FRANCESCO PARIBELLO, ANTONIO MACCIÒ

**Affiliations:** 1 Department of Medical Science ‘Mario Aresu’, University of Cagliari, Monserrato, Cagliari I-09042, Italy; 2 Medical Oncology Unit, ‘N.S. Bonaria’ Hospital, San Gavino I-09037, Italy; 3 Department of Obstetrics and Gynaecology, Sirai Hospital, Carbonia I-09013, Italy; 4 Department of Public Health, University of Cagliari, Monserrato, Cagliari I-09042, Italy; 5 Department of Gynecologic Oncology, ‘A. Businco’ Hospital, Regional Referral Center for Cancer Disease, Monserrato, Cagliari I-09121, Italy

**Keywords:** estrogen-dependent breast cancer, tamoxifen, luteinizing hormone-releasing hormone, ovarian cyst, estradiol, endometrial hyperplasia

## Abstract

Adjuvant endocrine therapy is an integral component of care for endocrine-dependent breast cancer. The aim of this type of therapy is to counteract the production and the action of estrogens. The ovary is the primary site of estrogen production in premenopausal women, whereas, in postmenopausal women, the main source of estrogens is adipose tissue. Therefore, ovarian function suppression is an effective adjuvant strategy in premenopausal estrogen-dependent breast cancer. Similarly, the inhibition of estrogen action at the receptor site by tamoxifen has proven to be effective. To date, international consensus statements recommend tamoxifen (20 mg/day) for five years as the standard adjuvant endocrine therapy for premenopausal women. It should be noted that tamoxifen is a potent inducer of ovarian function and consequent hyperestrogenism in premenopausal women. In the present study, we report two cases of ovarian cyst formation with very high estrogen levels and endometrial hyperplasia during the administration of tamoxifen alone as adjuvant treatment for estrogen receptor-positive breast cancer in premenopausal women. These cases suggest that in young premenopausal patients with estrogen-dependent breast cancer, ovarian suppression is an essential prerequisite for an adjuvant endocrine therapy with tamoxifen. In this context, luteinizing hormone-releasing hormone agonist treatment by suppressing effective ovarian function may lead to a hypoestrogenic status that may positively impact breast cancer prognosis and prevent the effects of tamoxifen at the gynecological level. It is important to reconsider the action of tamoxifen on ovarian function and include these specific effects of tamoxifen in the informed consent of premenopausal patients who are candidates for tamoxifen alone as adjuvant endocrine treatment.

## Introduction

Adjuvant endocrine therapy is an integral component of care for endocrine-dependent breast cancer (EDBC). The goal of this type of therapy is to counteract the production and the action of estrogens. The ovary is the primary site of estrogen production in premenopausal women, whereas, in postmenopausal women, the main source of estrogens is adipose tissue. Therefore, ovarian function suppression [by surgery, radiotherapy, chemotherapy and luteinizing hormone-releasing hormone (LHRH) agonists] is an effective adjuvant strategy in premenopausal women with EDBC. Similarly, the inhibition of estrogen action at the receptor site by tamoxifen has proven to be effective (1).

To date, international consensus statements recommend tamoxifen (20 mg/day) for five years as the standard adjuvant endocrine therapy for premenopausal women, while the role of LHRH agonists remains controversial and under active investigation (2–4). In particular, the value of the addition of ovarian suppression by LHRH agonists along with tamoxifen, particularly in chemotherapy-treated patients who may develop ovarian failure as a consequence of cytotoxic treatment, is not well-defined (2,4–7).

It should be noted that tamoxifen is a potent inducer of ovarian function in premenopausal women (8). The evaluation of endocrine parameters in premenopausal women during treatment with tamoxifen as a single agent has demonstrated that the levels of estradiol, estrone and progesterone are elevated one- to three-fold (9). Therefore, the effect of numerous years of ovarian stimulation by tamoxifen must be evaluated, particularly in women with node-negative disease or in healthy women in whom tamoxifen is used to prevent breast cancer. Equally important is the effect that the hyperestrogenism induced by tamoxifen exerts at the endometrial level (10,11).

In the present study, we report two cases of ovarian cyst formation and endometrial hyperplasia induced by tamoxifen used alone as adjuvant treatment for estrogen positive breast cancer in premenopausal women. This study demonstrated the requirement for the administration of LHRH agonist to effectively suppress the tamoxifen-induced estrogen hyperproduction by the ovaries. Patients provided written informed consent.

## Case reports

### Case 1

In January 2013, a 37-year-old woman was admitted to the Department of Obstetrics and Gynaecology, Sirai Hospital (Carbonia, Italy) with a diagnosis of bilateral ovarian cysts associated with endometrial hyperplasia and lower abdominal pain, with suspected ovarian malignancy. The patient reported amenorrhea and was willing to have a pregnancy.

Two years previously, the patient had undergone conservative breast surgery for high-grade ductal carcinoma of the right breast [stage I; estrogen receptor (ER)-positive, 90%; progesterone receptor (PgR)-positive; 80%; human epidermal growth factor 2-negative; Ki67 labeling index, 10%]. Following surgery, the patient received breast irradiation and adjuvant tamoxifen therapy without an LHRH agonist. There was no history of ovarian enlargement prior to tamoxifen administration.

**Figure 1:**
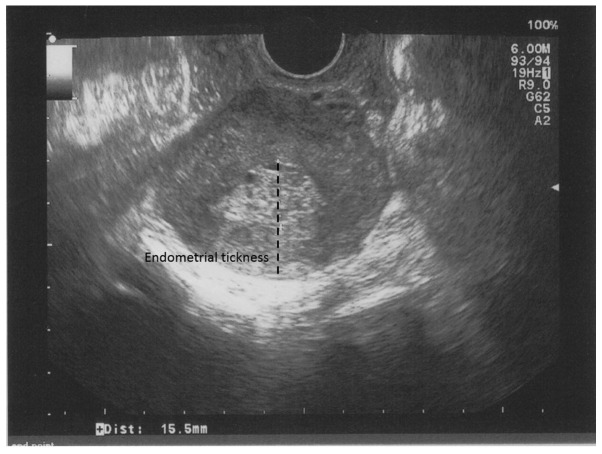
Case 1: Endometrial hyperplasia. Transvaginal ultrasonography at admission showed an endometrial hyperplasia with a hyperechogenic heterogeneous endometrial pattern with a thickness of 15.5 mm.

**Figure 2:**
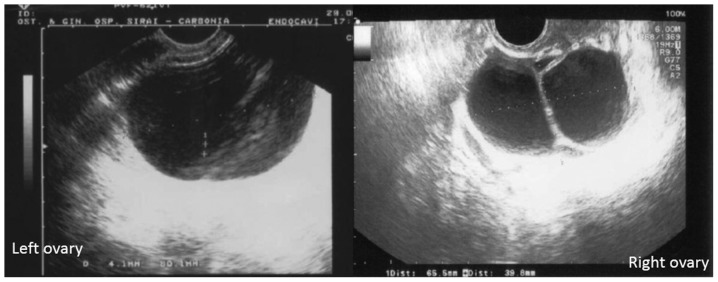
Case 1: Bilateral ovarian cysts. Transvaginal ultrasonography examination at admission showed a right multilocular ovarian cyst (85×40 mm) and a left multiloculated mass (65×46 mm).

During the periodic oncological follow-up examinations, a transvaginal sonogram demonstrated endometrial hyperplasia with a hyperechogenic heterogeneous endometrial pattern with a thickness of 15.5 mm (Fig. 1). Bilateral ovarian cysts were also observed, including a right multilocular ovarian cyst (85×40 mm) and a left multiloculated mass (65×46 mm). Color Doppler sonography showed partially vascularized intracystic septa (Fig. 2). No evidence of ascites was observed and tumor markers, cancer antigen (CA)-125, CA-15.3 and carcinoembryonic antigen (CEA), were within the normal range.

As the patient was undergoing tamoxifen treatment without ovarian suppression with a LHRH agonist, we hypothesized that ovarian hyperstimulation was present. Subsequently, the levels of serum estradiol were measured and identified to be 1,200 pg/ml. The patient was symptomatic with lower abdominal pain and, thus, laparoscopic bilateral ovarian cystectomy was performed. The extemporaneous histological examination revealed bilateral follicular ovarian cysts. A hysteroscopy with biopsy was also performed, and the histological examination showed a ‘simplex endometrial hyperplasia’. The postoperative course was without complications, and the patient was discharged two days later.

Continuation of tamoxifen therapy plus the addition of an LHRH agonist was discussed with the patient, and the patient accepted. The pelvic and transvaginal ultrasound (US) examination three months later showed a regression of the endometrial hyperplasia (thickness, 7.6 mm). The patient has continued the adjuvant treatment with tamoxifen and an LHRH agonist, and the follow-up examinations of one year to February 2014 have been negative for breast cancer relapse. Transvaginal US evaluation over one year after surgery showed an endometrial thickness in the normal range (<8 mm), normal ovaries (left ovary, 33×17 mm in the largest sagittal diameter; right ovary, 24×10 mm in the perpendicular diameter) and estradiol levels (<20.00 pg/ml).

### Case 2

In June 2013, a 33-year-old woman was admitted to the Department of Gynaecologic Oncology, A. Businco Hospital (Cagliari, Italy) with a diagnosis of left ovarian cysts and endometrial hyperplasia. Two years previously, the patient underwent radical mastectomy plus ipsilateral lymphadenectomy for a low grade papillary carcinoma of the right breast (stage I; ER-positive, 80%, PgR-positive, 80%; HER2-negative, Ki67 labeling index, 15%). Following surgery, adjuvant endocrine treatment with tamoxifen and an LHRH agonist was initiated.

After 10 months, the patient chose to terminate the LHRH agonist treatment for self-reported side effects consisting of insomnia, irritability and arthralgia. The patient continued to receive 20 mg/day of tamoxifen alone. At the 12th month following LHRH interruption, during the planned periodic examinations, a transvaginal US showed a heterogeneous endometrial pattern (thickness, 14 mm) and the presence of a multilocular left ovarian cyst (54×44×40 mm). Color duplex sonography showed no increased vascularization. No evidence of ascites was observed and laboratory data indicated an elevated serum estradiol concentration of 698.80 pg/ml. Tumor markers (CA-125, CA-15.3 and CEA) were within the normal range.

Continuation of tamoxifen therapy and the resumption of a LHRH analog were discussed with the patient, and the patient accepted. The pelvic and transvaginal US examination three months later showed an endometrial hyperechogenic pattern with a thickness of 7.6 mm, and normal ovaries (left, 20×15.9 mm in the largest sagittal diameter; right, 21×16 mm in the perpendicular diameter). The patient’s estradiol levels decreased to 22.14 pg/ml. The next follow-up assessments to February 2014 showed normal endometrial thickness, ovaries and estradiol levels; in addition, examinations for breast cancer recurrence were negative.

## Discussion

The cases described in the present report demonstrated the presence of functional ovarian cysts with very high estrogen levels during the administration of tamoxifen alone as an adjuvant treatment for premenopausal EDBC. The patients also presented with biopsy-proven endometrial hyperplasia.

A limited number of studies have reported cases of tamoxifen-induced ovarian cysts in breast cancer patients (8,12–17). These papers show that tamoxifen-induced ovarian cysts commonly occur after three months of tamoxifen treatment, with the highest incidence in the interval between three to 11 months after treatment initiation. Additionally, the development of ovarian cysts after two years of tamoxifen treatment is extremely rare.

Tamoxifen therapy for five years is considered the standard endocrine therapy for premenopausal women with EDBC (18). However, data on the impact of tamoxifen on ovarian function are often lacking in the literature. As the ovary is the main source of estrogen in premenopausal women, the evaluation of ovarian function during tamoxifen treatment should represent a central issue in the management of EDBC in premenopausal women. It has been reported that during tamoxifen treatment, a percentage of premenopausal patients have increased ovarian function associated with elevated estradiol levels. In these patients, the amenorrhea, if present, may falsely suggest ovarian failure, masking the presence of hyperactive ovaries (19).

Tamoxifen may increase plasma estrogen concentrations by interfering with normal negative pituitary feedback mechanisms, with a resulting increase in follicle-stimulating hormone-driven ovarian steroidogenesis (12). Subsequently, the development of ovarian cysts associated with high estradiol levels indicates the presence of hyperactive ovaries as a consequence of tamoxifen action. An additional mechanism involved in the increased estrogen production by tamoxifen is its direct effect on granulosa cells (20).

It is still currently debated whether the ovarian stimulation induced by tamoxifen could theoretically interfere with its antitumoral effects in premenopausal EDBC. In this context, a high-priority research question is whether additional benefit is gained with the use of LHRH agonists in addition to tamoxifen or as an alternative (21). Two meta-analyses of randomized clinical trials assessing the role of LHRH agonists in the adjuvant treatment of premenopausal EDBC patients (22,23) demonstrated a clear benefit in terms of recurrence rate and disease-free survival from ovarian suppression with LHRH agonists, both in combination with tamoxifen and as a single intervention. Notably, when assessing the subgroup of patients undergoing adjuvant chemotherapy that is expected to induce a menopausal status, a significant benefit in terms of reduced recurrence rate and death has been observed only in very young premenopausal women (aged ≤40 years) (23). It is hypothesized that chemotherapy is less likely to induce permanent amenorrhea in this population of patients than in older women. This evidence may be even more significant in women receiving more modern based chemotherapy regimens, which induce less commonly permanent amenorrhea (24), and even more significant in premenopausal patients who are not candidates for adjuvant chemotherapy. In this context, noteworthy data have been presented by Mourits *et al* (8), who showed that in patients who remained premenopausal after standard dose chemotherapy, tamoxifen use was associated, despite amenorrhea, with the development of ovarian cysts associated with the high estradiol levels that were indicative of overactive ovaries.

The findings of the present study suggested that, in addition to the concerns regarding the optimum endocrine adjuvant treatment for premenopausal breast cancer, the effect of tamoxifen on the endometrium should be carefully considered. Ovarian hyperstimulation, with increasing circulating estrogens, induced by tamoxifen in premenopausal patients, may significantly influence the occurrence of endometrial hyperplasia and the subsequent risk of endometrial cancer. Additionally, the direct proliferative effect of tamoxifen on the endometrium should be considered (11,25).

Thus, in young premenopausal patients with estrogen-dependent breast cancer, ovarian suppression is an essential prerequisite for an adjuvant endocrine with tamoxifen. In this context, LHRH agonist treatment by suppressing effective ovarian function may lead to a hypoestrogenic status that may positively impact breast cancer prognosis (23) and prevent the effects of tamoxifen at the gynecological level (endometrial hyperplasia and ovarian cyst formation) (26). In the literature, the majority of reported tamoxifen-induced ovarian cysts disappeared following cessation of tamoxifen treatment (13). In addition, cotreatment with tamoxifen and an LHRH agonist resolved ovarian cysts (14–17). By contrast, expectant management without abandoning tamoxifen use may cause complications, such as torsion and cystic necrosis; in these latter cases, even if the ovarian enlargement is benign, the growth of cysts may require surgical intervention with an increased risk of morbidity.

In conclusion, based on this evidence, it is important to reconsider the action of tamoxifen on ovarian function, and include these specific effects of tamoxifen on ovarian activity in the informed consent of premenopausal patients who are candidates for tamoxifen alone as adjuvant endocrine treatment.
